# Predicting future cognitive decline from non-brain and multimodal brain imaging data in healthy and pathological aging

**DOI:** 10.1016/j.neurobiolaging.2022.06.008

**Published:** 2022-06-28

**Authors:** Bruno Hebling Vieira, Franziskus Liem, Kamalaker Dadi, Denis A. Engemann, Alexandre Gramfort, Pierre Bellec, Richard Cameron Craddock, Jessica S. Damoiseaux, Christopher J. Steele, Tal Yarkoni, Nicolas Langer, Daniel S. Margulies, Gaël Varoquaux

**Affiliations:** a Methods of Plasticity Research, Department of Psychology, University of Zurich, Zurich, Switzerland; b Neuroscience Center Zurich (ZNZ), University of Zurich & ETH Zurich, Zurich, Switzerland; c University Research Priority Program “Dynamics of Healthy Aging”, University of Zurich, Zurich, Switzerland; d UniversitéParis-Saclay, Inria, CEA, Palaiseau, France; e Department of Neurology, Max Planck Institute for Human Cognitive and Brain Sciences, Leipzig, Germany; f Functional Neuroimaging Unit, Geriatric Institute, University of Montreal, Montreal, Quebec, Canada; g Computational Neuroimaging Lab, Dell Medical School, The University of Texas, Austin, TX, USA; h Institute of Gerontology and the Department of Psychology, Wayne State University, Detroit, MI, USA; i Department of Psychology, Concordia University, Montreal, Canada; j Cognitive Neuroanatomy Lab, Institut du Cerveau et de la Moelle épinière, Paris, France; k Department of Psychology, The University of Texas, Austin, TX, USA

**Keywords:** Biomarker, Machine learning, Predictive modeling, Cross-validation, Open science

## Abstract

Previous literature has focused on predicting a diagnostic label from structural brain imaging. Since subtle changes in the brain precede a cognitive decline in healthy and pathological aging, our study predicts future decline as a continuous trajectory instead. Here, we tested whether baseline multimodal neuroimaging data improve the prediction of future cognitive decline in healthy and pathological aging. Nonbrain data (demographics, clinical, and neuropsychological scores), structural MRI, and functional connectivity data from OASIS-3 (N = 662; age = 46–96 years) were entered into cross-validated multitarget random forest models to predict future cognitive decline (measured by CDR and MMSE), on average 5.8 years into the future. The analysis was preregistered, and all analysis code is publicly available. Combining non-brain with structural data improved the continuous prediction of future cognitive decline (best test-set performance: R2 = 0.42). Cognitive performance, daily functioning, and subcortical volume drove the performance of our model. Including functional connectivity did not improve predictive accuracy. In the future, the prognosis of age-related cognitive decline may enable earlier and more effective individualized cognitive, pharmacological, and behavioral interventions.

## 1. Introduction

Cognitive decline, such as worsening memory or executive functioning, occurs in healthy and pathological aging. Crucially, the noticeable decline may be preceded by subtle changes in the brain. It is this sequence that enables using brain imaging data to predict the current cognitive functioning of a person or related surrogate markers. For example, structural brain imaging has been used to predict patients’ current cognitive diagnosis (Rathore et al., 2017), or brain age (Cole and Franke, 2017), a surrogate biomarker related to cognitive impairment (Liem et al., 2017). Together, these findings demonstrate the clinical potential of neuroimaging data used in combination with predictive analyses.

While predicting *current* cognitive functioning enables insight into related brain markers, predicting *future* cognitive decline from baseline data poses a greater challenge with more substantial clinical relevance (Davatzikos, 2019). Using current brain imaging data to predict a current diagnostic label (such as dementia), targets a label that can fairly easily be determined via other means such as clinical assessments (and usually with less cost than brain imaging). When predicting future cognitive change, however, brain imaging might aid a prognosis with greater clinical utility that cannot be easily obtained otherwise. Most previous studies that predicted future change restricted their analysis to whether patients with mild cognitive impairment (MCI) converted to Alzheimer’s disease (AD) (e.g., Eskildsen et al., 2015; Korolev et al., 2016; Gaser et al., 2013; Davatzikos et al., 2011) or predicted membership in data-driven trajectory-groups of future decline (Bhagwat et al., 2018). See Table 1 for a comparison. Predicting future cognitive decline on a continuum (instead of forming distinct diagnostic labels from cognitive data) better characterizes the underlying change in abilities on an individual level. This approach can also be used to widen the scope of applications by including healthy aging. Brain data is a rich source of information that might help us better understand and even reorganize diagnostic syndromes or categories.

While most previous predictive studies used structural brain imaging alone, integrating structural and functional imaging has been shown to improve predictions. Since both brain structure (Oschwald et al., 2019) and brain function (Liem et al., 2020) change in aging, the most accurate predictions of brain age have come from combining them (Liem et al., 2017; Engemann et al., 2020; Schulz et al., 2022). Multimodal gains have also been shown in more complex predictions such as current diagnosis in AD (Rahim et al., 2016) and conversion from MCI to AD (e.g., Hojjati et al., 2018; Dansereau et al., 2017; Tam et al., 2019). Therefore, integrating multiple brain imaging modalities enables more complete characterization of brain aging and provides increased predictive power.

Demographic, health, and clinical variables, which are straightforward to obtain and non-invasive, were demonstrated to reliably predict the conversion to cognitive impairment in elders over a 2-year period (Na, 2019) in the absence of any brain imaging data, demonstrating the value of nonbrain data. Likewise, nonbrain data pertaining to mood, demographics, lifestyle, education, and early-life factors were shown to be on par with brain imaging data in the prediction of intelligence and neuroticism, but not brain-age delta (Dadi et al., 2021).

The present study aimed to predict future cognitive decline from baseline data in healthy and pathological aging. We combined nonbrain data, such as scores from clinical assessments and demographics, with multimodal brain imaging data to test whether adding brain imaging to nonbrain data improves predictive performance, and whether multimodal imaging outperforms single imaging modalities. We showed that structural imaging in particular improved continuous prediction of future cognitive decline. An early prognosis of future cognitive decline might enable earlier and more effective pharmacological or behavioral treatments to be tailored to the individual, resulting in more efficiently allocated medical resources.

## 2. Methods

The analysis presented here was preregistered (Liem et al., 2019). We largely followed this preregistration and deviations are described in the supplement (*6.1.2 Deviation from preregistration*). The deviations concern minor details in data analysis and do not affect the qualitative conclusions we draw. Additionally, we performed nonpreregistered validation analyses that were suggested by the main results.

### 2.1. Sample and session selection

The present analysis aimed to predict future cognitive decline from baseline nonbrain (e.g., age and clinical scores) and brain imaging data (regional brain volume and functional connectivity). We used data from the publicly available, longitudinal OASIS-3 project, a collection of data from several studies at the Washington University Knight Alzheimer Disease Research Center (LaMontagne et al., 2019). OASIS-3 acquired data in different types of sessions (*clinical sessions:* nonbrain data describing personal characteristics, cognitive and everyday functioning, health; *neuropsychological sessions:* nonbrain data from neuropsychological tests; *MRI sessions:* structural and functional MRI). The count and spacing between sessions varied between participants. To predict future cognitive decline, baseline sessions were used as input data and follow-up sessions as targets. The study design required a matching approach to select: (1) baseline sessions (from clinical, neuropsychological, and MRI sessions) to be used as input data, and (2) follow-up clinical sessions to estimate the future cognitive decline.

First, baseline data were established by matching sessions from the different types (clinical, neuropsychological, MRI). We matched each MRI session that had at least 1 T1w and 1 fMRI scan with the closest clinical session. For each participant, the first MRI-clinical-session pair with an absolute time difference of < 1 year was selected as the baseline session. If no such pair was available, the participant was excluded from the analysis. Additionally, the closest neuropsychological session (within 1 year of the MRI baseline session) was also considered as baseline data. Baseline information from neuropsychological testing, however, was considered optional, and not finding a matching neuropsychological session was not a criterion for exclusion. All data preceding the selected baseline sessions were disregarded for the analysis.

Second, all clinical sessions after the baseline clinical session were included as follow-up sessions to estimate cognitive decline. To reliably estimate decline, participants were only included if they had at least 3 clinical sessions (baseline plus 2 follow-up sessions). This matching approach reduced the sample (N_total_ = 1098) to 662 participants (302 male; Table 2).^1^ The majority were cognitively healthy at baseline (509 healthy controls, 12 were diagnosed with MCI, and 111 with dementia; for 30 no diagnosis was available for the baseline session).

MRI data was downloaded in BIDS format (K. J. Gorgolewski et al., 2016) via scripts provided by the OASIS project.^2^ Nonbrain data was downloaded via XNAT central.^3^

### 2.2. Data

#### 2.2.1. Nonbrain data

Non-brain data described personal characteristics at baseline, such as demographics, cognitive, and everyday functioning, genetics, and health (Table 3 shows the abbreviations of tests). For further information on the measurements, see relevant publications by the OASIS team (LaMontagne et al., 2019; Morris et al., 2006; Weintraub et al., 2009).

The specific measures included:

1. Demographic information: sex, age, education
2. clinical scores: MMSE (Folstein, Folstein, and McHugh, 1975), CDR (Morris, 1993), FAQ (Jette et al., 1986), NPI-Q (Kaufer et al., 2000), GDS (Geriatric Depression Scale, Yesavage et al., 1982)
3. neuropsychological scores: WMS-R (Elwood, 1991), Word fluency, TMT (Bucks, 2013), WAIS-R (Franzen, 2000), BNT (Borod, Goodglass, and Kaplan, 1980)
4. APOE genotype
5. a cognitive diagnosis (healthy control, MCI, dementia)
6. health information: cardio/cerebrovascular health, diabetes, hypercholesterolemia, smoking, family history of dementia
7. the number of clinical sessions conducted before the selected baseline session (for instance sessions without a matching MRI session) to account for retest effects

#### 2.2.2. MRI data

MRI data were acquired on Siemens 3T scanners, with the majority coming from a TrioTim model (622 of 662 participants), and the rest from the combined PET/MRI Biograph mMR model. Each participant had between 1 and 4 T1w scans (1.7 on average). In total, the sample had 1′119 T1w images. The parameter combination most used (in over 1′070 scans) was voxel size = 1 × 1 × 1 mm^3^, echo time (TE) = 0.003 second, repetition time (TR) = 2.4 seconds. Where available, T2w images were also used to aid surface reconstruction. In total, 618 participants had a T2w image. The parameters for the T2w images were voxel size = 1 × 1 × 1 mm^3^, TE = 0.455 second, TR = 3.2 seconds.

Each participant had between 1 and 4 functional resting-state scans (M = 2.0). In total, the sample had 1′327 functional images. The parameter combination most used (in over 1′300 scans) was voxel size = 4 × 4 × 4 mm^3^, TE = 0.027 second, TR = 2.2 seconds, scan duration = 6 minutes. For further information regarding the imaging data see (LaMontagne et al., 2019).

See Table S1 for complete information about T1w, T2w and fMRI scans acquisitions.

### 2.3. MRI preprocessing

Functional and structural MRI data were preprocessed using the standard processing pipeline of *fMRIPrep* 1.4.1 (Esteban et al. 2018), which also includes running *FreeSurfer* 6.0.1 on the structural images (Fischl, 2012). A detailed description of the preprocessing can be found in the supplement (*6.1.1 Details on MRI preprocessing*). Except for basic validity checks in a random subset of participants, data quality of the preprocessed data was not rigorously assessed. Notably *fMRIPrep* has been shown to robustly work across many datasets (Esteban et al. 2018).

### 2.4. Feature extraction

Input data from nonbrain and brain imaging modalities at baseline were used to predict future cognitive decline (predictive targets). In the following sections we provide further details on the features that entered the predictive models.

#### 2.4.1. Input data

**Fig. 1.**
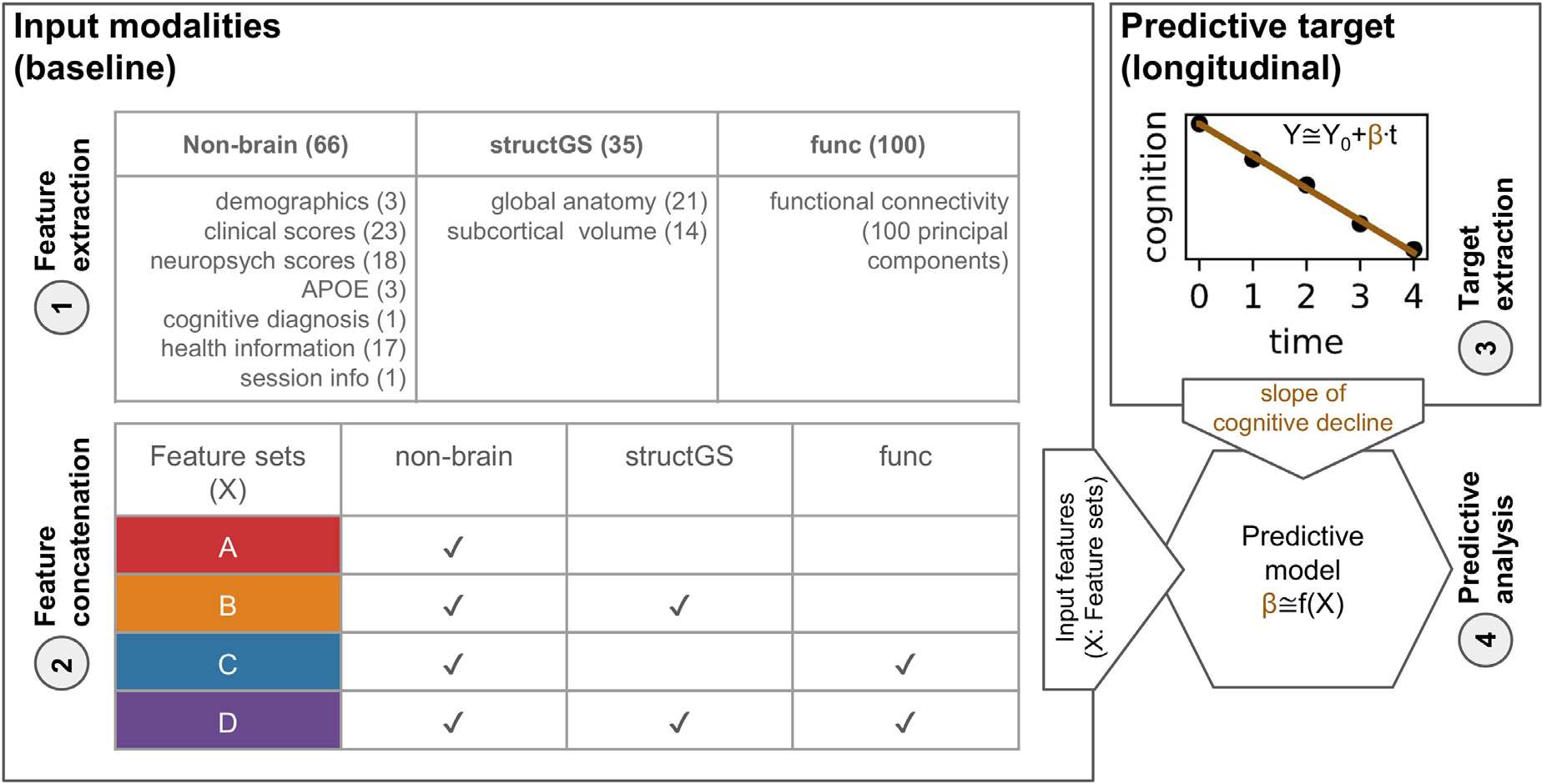
Overview of the predictive approach. (A) Features from non-brain, structGS (global and subcortical structural), and func (functional connectivity) modalities are extracted from baseline data. The number of features is provided in parentheses. (B) Feature concatenation produces sets of multimodal input features. For instance, red represents nonbrain features only, while orange represents a combination of nonbrain and structGS. (C) Extraction of slopes representing a cognitive change from CDR (Clinical Dementia Rating) and MMSE (Mini-Mental State Examination). (D) Models are trained to predict cognitive decline based on the input features. Here, we used a multitarget random forest model within a nested cross-validation approach to predict CDR and MMSE change simultaneously.

**Fig. 2.**
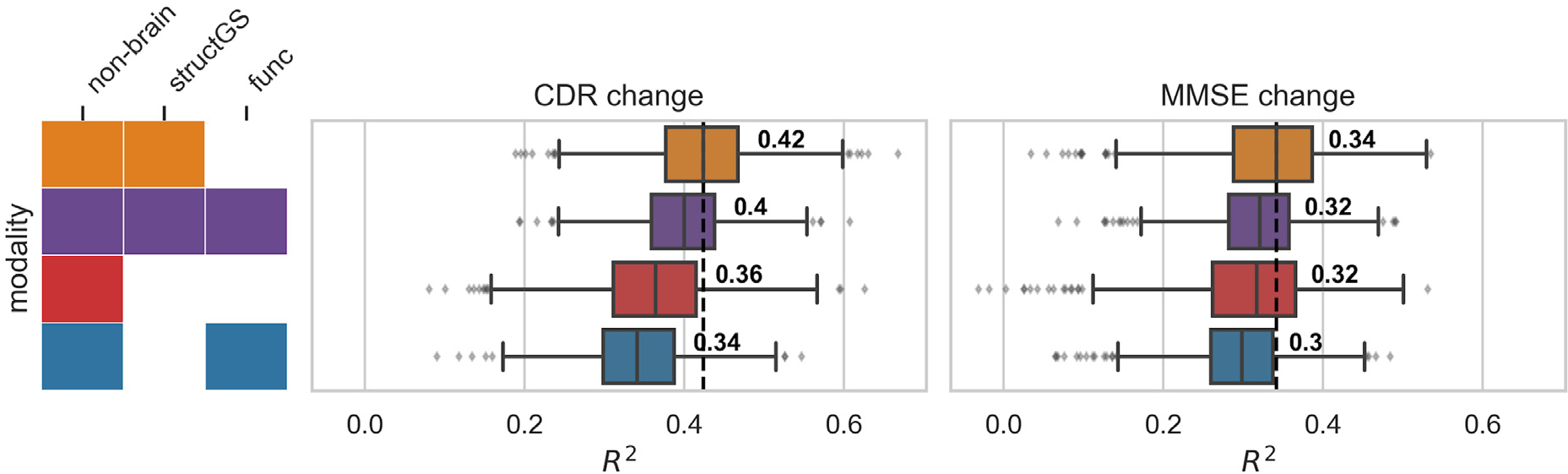
Adding structural data (orange) to nonbrain data (red) improved the prediction of cognitive decline. Test performance (R2, coefficient of determination, x-axis) across splits (Nsplits = 1000) for the combinations of input modalities (y-axis). Targets: cognitive change measured via CDR (Clinical Dementia Rating, middle) and MMSE (Mini-Mental State Examination, right). Input modalities: non-brain, structGS (global and subcortical structural volumes), func (functional connectivity). The left panel represents combinations of input modalities (e.g., orange is nonbrain + structGS). The number represents the median, the dashed vertical line marks the median of the best-performing combination of modalities (within a target measure). For the full results that include single-modality brain imaging, see Figure S2.

Input data for the predictive models came from 3 modalities: *non-brain*, global and subcortical structural (*structGS*), and functional connectivity (*func*; Fig. 1A). Modalities were entered into the models on their own and in combination. For instance, *nonbrain + structGS* models received horizontally concatenated input features from the *nonbrain* and *structGS* modalities (Fig. 1 and 2). This allowed testing whether combining nonbrain with structural data improved predictive accuracy as compared to nonbrain data alone. The following paragraphs describe the input data modalities and Table S2 gives an overview of features entered into the models.

##### 2.4.1.1. Nonbrain data.

Nonbrain features included demographics, scores of clinical and neuropsychological instruments, APOE genotype, and health information. For a detailed list see Table S2. In total, 66 features entered the models from the *nonbrain* modality.

##### 2.4.1.2. Structural MRI (structGS).

For the *structGS* modality (global and subcortical structure), anatomical markers were extracted from the *FreeSurfer*-preprocessed anatomical scans. Following our previous work (Liem et al., 2017), we extracted global structural markers (volume of cerebellar and cerebral GM and WM, subcortical GM, ventricles, corpus callosum, and mean cortical thickness) and the volumes of 7 subcortical regions (accumbens, amygdala, caudate, hippocampus, pallidum, putamen, thalamus; for each hemisphere separately). Most markers were extracted from the *aseg* file, except for mean cortical thickness, which was extracted from the *aparc.a2009s* parcellation (Desikan et al., 2006). To account for head-size effects, volumetric values were normalized by estimated total intracranial volume. In total, 35 features entered the models from the *structGS* modality.

##### 2.4.1.3. Functional MRI (func).

Functional connectivity was computed from the *fMRIPrep*-preprocessed functional scans. Denoising was performed using the 36P model (Ciric et al., 2017), which includes signals from 6 motion parameters, global, white matter, and CSF signals, derivatives, quadratic terms, and squared derivatives. Time series were extracted from 300 cortical, cerebellar, and subcortical coordinates of the Seitzman atlas (Seitzman et al., 2020) using balls of 5 mm radius. The signals were band-pass filtered (0.01–0.1 Hz) and linearly detrended. Connectivity matrices were extracted by correlating the time series using Pearson correlation and applying Fisher-z-transformation. If multiple fMRI runs were available, the z-transformed connectivity matrices were averaged within participants. The vectorized upper triangle of this connectivity matrix was entered into the predictive pipeline and was further downsampled to 100 PCA components within cross-validation (see below). Denoising and feature extraction was performed with *Nilearn* 0.6.0 (Abraham et al., 2014).

Due to the dimensionality of connectivity matrices, we opted to perform PCA-based dimensionality reduction on the fMRI data. In its raw form, the fMRI connectivity has 44,850 unique features, a prohibitive amount that eclipses the other modalities. Because trees are grown sampling both data points and variables, this number would complicate the training of Random Forests, requiring an increase in the number of trees to reliably expose the different modalities to the model. This problem is alleviated by reducing this feature set to 100 components. Because the degrees of freedom are greatly decreased, it also reduces the risk of overfitting. This solution has been successfully applied in many examples in the literature. Schulz et al., (2022), for example, use PCA so that comparisons between modalities and modality combinations use the same number of features.

#### 2.4.2. Predictive targets

**Fig. 3.**
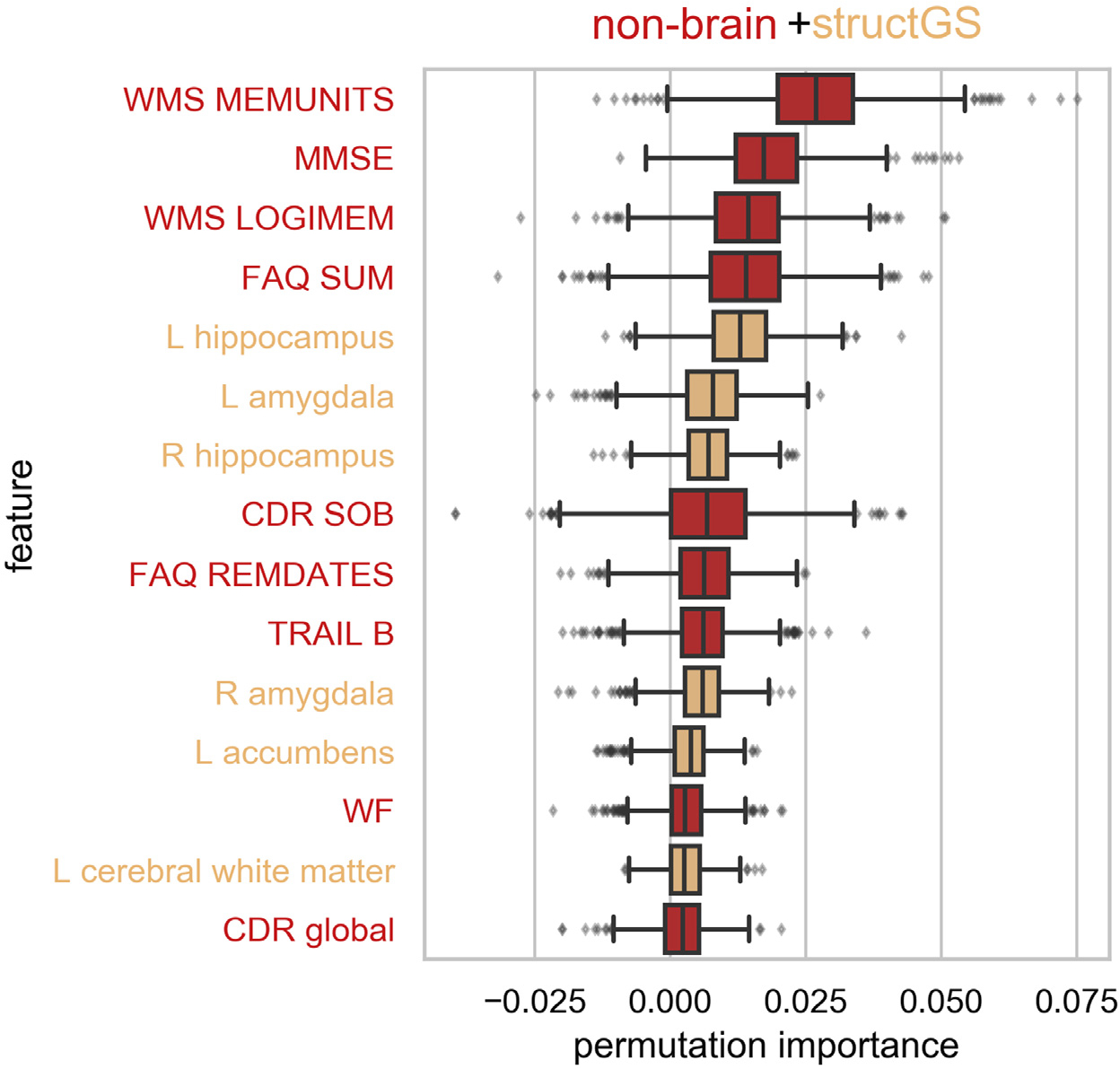
Cognitive performance, daily functioning, and subcortical volume were among the most informative features. Permutation importance of the top 15 features of the nonbrain + structGS model (median across splits). Permutation importance is quantified as the decrease in test performance R2 with the feature permuted. Red: nonbrain features, light orange: structGS features. CDR, clinical dementia rating; FAQ, functional assessment questionnaire; L, left; MMSE, mini-mental state examination; LOGIMEM, Total number of story units recalled from this current test administration; MEMUNITS, Total number of story units recalled (delayed); R, right; REMDATES, difficulty remembering dates; SOB, sum of boxes; SOB, sum of boxes score; TRAIL B, trail making test B; WF, word fluency, WMS, Wechsler memory scale.

To quantify future cognitive decline, trajectories of 2 clinical assessments, the CDR (Clinical Dementia Rating, Sum of Boxes score) and the MMSE (sum score of the Mini-Mental State Examination) were estimated using an ordinary least squares linear regression model for each participant and assessment independently (Fig. 1–3; for information on the count and timing of sessions, see Table 2). A linear slope was fitted through the raw scores of the follow-up session with the intercept fixed at the raw score of the baseline session (*score*_*foll ow*–*up*, *assessment*_ ~ *score*_*baseline*, *assessment*_ + *β*_*slope*, *assessment*_ × *time*). This approach was chosen over a linear mixed-effects model, as the mixed-effects model requires data from multiple participants, making cross-validation more convoluted. The resulting 2 parameters (*β*_*slope*, *CDR*_ and *β*_*slope*, *MMSE*_) were the 2 targets that were simultaneously predicted in the predictive analysis using a multitarget approach (Rahim et al., 2017). Slopes were estimated with *Statsmodels* 0.10.1 (Seabold and Perktold, 2010). The distribution of the estimated targets is plotted in Figure S1.

Two factors were fundamental to the adoption of the CDR and MMSE slopes as predictive targets. First, the number of participants with CDR and MMSE baseline scores is slightly higher than the number of participants with FAQ and NPI-Q scores. See Table 2. Second, both the CDR and MMSE are widely regarded as reliable tests for the clinical assessment and staging of dementia. FAQ, for example, is a questionnaire designed for bedside assessment and research based on instrumental activities of daily life (Pfeffer et al., 1982), which entails a degree of subjectivity, and NPI-Q is a brief informant-based questionnaire for general neuropsychiatric assessment (Kaufer et al., 2000). We believe the CDR and MMSE were the best candidates as specific measures of cognitive status for these 2 reasons.

### 2.5. Predictive analysis

**Fig. 4.**
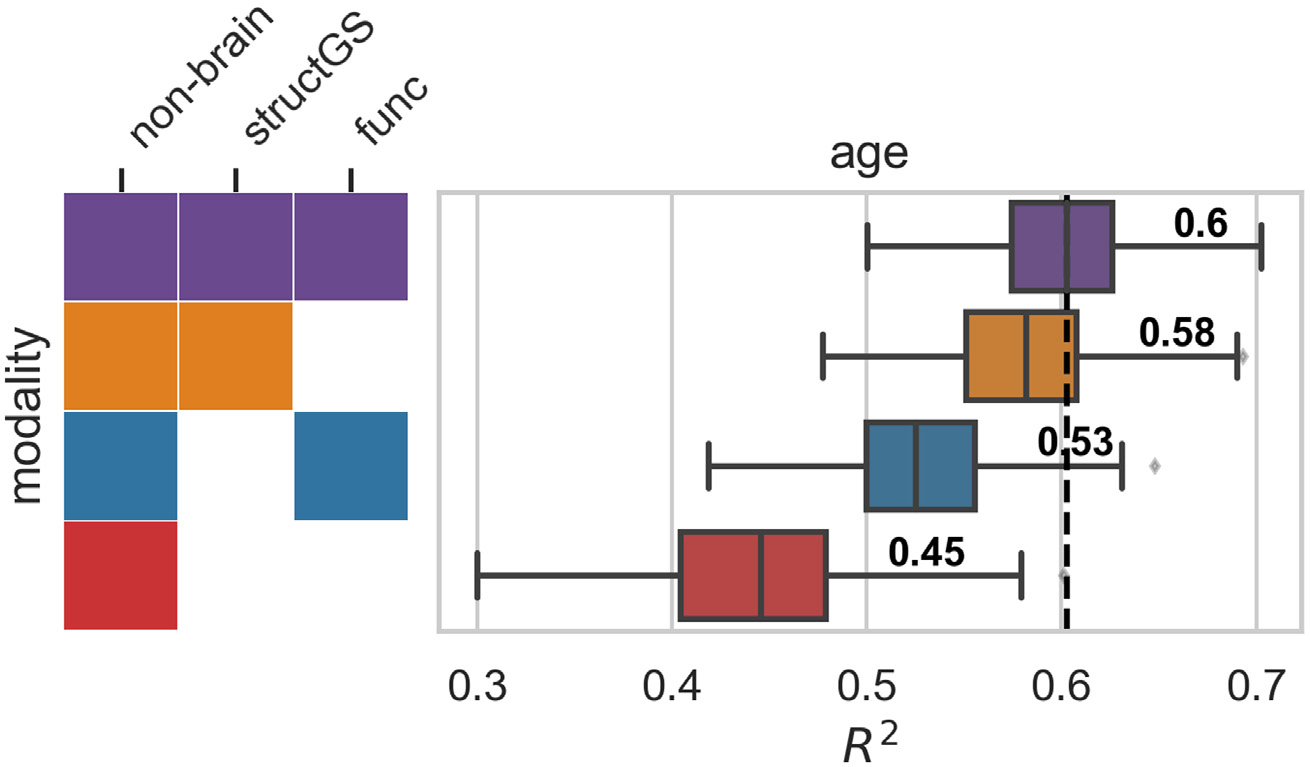
Multimodal imaging improves brain age prediction. Input modalities: non-brain, structGS (global and subcortical structural volumes), func (functional connectivity). The number represents the median, the dashed vertical line marks the median of the best-performing combination of modalities. For the full results that include single-modality brain imaging, see Figure S6.

The predictive pipeline (Fig. 1–4) consisted of a multivariate imputer (*Scikit-learn’s* IterativeImputer) (Buck, 1960) and a multitarget random forest (RF) regression model (Breiman, 2001). Multivariate imputation has recently been shown to work in combination with predictive models in different missingness scenarios (Josse et al., 2019). It comprises using all other input variables to estimate missing values in each input variable vector. The procedure is then repeated now including the previously imputed values as inputs for a number of iterations. RF is a nonparametric machine-learning algorithm based on ensembles of decision trees. Trees are trained in parallel over bootstrap samples, also including the sampling of input variables. The final output is obtained by aggregating the outputs of a predetermined number of trees.

Predictive models were trained using nested cross-validation via a stratified shuffle-split (1000 splits, 80% training, 20% test participants, stratified by the targets). In the inner loop, the RF’s hyperparameters were tuned via grid search on the training participants (the tree depth was selected among [3, 5, 7, 10, 15, 20, 40, 50, None], where None leads to fully grown trees; the criterion to measure the quality of an RF-split was tuned with ‘mean squared error’ and ‘mean absolute error’). The best estimator was carried forward to determine its out-of-sample performance on the test participants. To derive an estimate of chance performance, null models were also trained and evaluated with permuted target values. For each cross-validation split, we calculated the coefficient of determination (R^2^) on the test predictions. All predictive analyses were performed using *Scikit-learn* 0.22.1 (Pedregosa et al., 2011).

Model comparison was used to determine whether 1 model offered better prediction accuracy than another (for instance, to check whether a given model outperformed the null model, or whether a model with added brain imaging data improved accuracy as compared to a model using only nonbrain data). Model comparison in cross-validation needs to take the dependence between splits into account, complicating statistical tests (Bengio and Grandvalet, 2004). Thus, instead of calculating a formal statistical test, we calculated the number of splits for which the model in question outperformed the reference model, resulting in a percent value, with numbers close to 100% denoting models which robustly outperformed the reference model (Engemann et al., 2020).

To inspect which features contributed to a prediction, permutation importance was calculated (Breiman, 2001). Permutation importance evaluates the effects of features on the predictive performance by permuting feature values. If shuffling a feature does decrease performance, it is considered important for the model. It must be noted that this approach might underestimate the importance of correlated features. This is attributable to the fact that when one of the features is permuted, the second 1 retains some of the information that both shared. For example, this might happen with baseline MMSE and CDR-SOB scores, which are correlated in the general population (Balsis et al., 2015), and are both used in the nonbrain data. Furthermore, learning curves were estimated to assess whether the number of participants in the analysis was sufficient. For these comparisons, models were trained with increasing sample size while observing the test performance.

We performed additional analyses to diagnose the predictive pipeline and present our results in context. First, to validate the pipeline and analysis code, the same predictive methodology was used to predict age, a strong and well-established effect (Liem et al., 2017). For this validation, age was removed from the input data and the approach followed in the main analysis was repeated using a ridge regression model and 200 cross-validation splits. All features were normalized for this analysis since ridge regression is sensitive to the variance of individual features. Ridge regression was selected for this analysis because research shows that linear models tend to perform on par with nonlinear models in age prediction (Schulz et al., 2020, 2022) and other benchmarks (Dadi et al., 2019), especially in the current sample size.

Second, to better compare our results with previous work that predicted decline using class labels, we repeated the original pipeline to classify extreme groups of participants that are cognitively stable vs. participants with cognitive decline using random forest classifiers and 200 cross-validation splits. Participants with CDR-SOB slopes > 0.25/y were labeled as declining (N = 156), and a randomly drawn equal number of participants without change in CDR-SOB (N = 366) were labeled as stable. The threshold of 0.25/y was selected based on statistical considerations: it is the mean of CDR-SOB slopes, as shown in Table 2 and it also corresponds to the 80th percentile, so choosing a higher threshold would result in a much smaller number of cases available.

### 2.6. Open science statement

All data used in the analysis are publicly available via the OASIS-3 project (LaMontagne et al., 2019). The analysis plan was preregistered (Liem et al., 2019). All preprocessing and analyses were performed in *Python* using open-source software and the code for preprocessing and predictive analysis is publicly available (Liem, 2020).^4^ Furthermore, a docker container that includes all software and code to reproduce the preprocessing and predictive analysis is also provided.^5^

## 3. Results

### 3.1. Predicting cognitive decline

A combination of nonbrain and structural data gave the best predictions of future cognitive decline. Adding structural data improved the prediction for both the CDR (median test performance R^2^ increased from 0.36 to 0.42; Fig. 2, red vs. orange; for a scatter plot showing true vs. predicted values, see Figure S3) and the MMSE (0.32–0.34), as compared to predictions from nonbrain data alone. This increase occurred in a large majority of splits (91% of splits for CDR, 78% for MMSE; Table S3). In contrast, adding functional connectivity features to non-brain features, or to *nonbrain + structGS* features, slightly decreased predictive performance.

To tune the RF models to the given problem, hyperparameters were optimized in a grid search approach. Tuning curves showed the results to be robust across a wide range of hyperparameter settings (Figure S4). Furthermore, learning curves demonstrated a sufficient sample size in the current setting (Figure S5).

The models consistently outperformed null models. Comparing the predictions against a null model with permuted predictions showed that most modalities outperformed chance-level in 100% of splits (Table S4). The predictions based on functional connectivity were an exception and outperformed null-models to a lesser degree (91% of splits for CDR, 73% for MMSE).

### 3.2. Features that predict cognitive decline

We used permutation importance to characterize the most predictive features of the best performing modality (nonbrain + structGS). Within the top-15 features, nonbrain included memory scores, the baseline scores of the targets (CDR, MMSE), and scores from the FAQ (functional assessment questionnaire). The structural features predominantly included subcortical regions (left and right hippocampus and amygdala, left accumbens; Fig. 3).

### 3.3. Validation analyses

Although functional connectivity models predicted cognitive decline poorly, functional data improved accuracy when predicting brain-age. Since functional connectivity alone did not predict cognitive decline well and did not increase the predictive accuracy of the nonbrain model (Figure S2), we conducted a validation analysis to ensure that our functional connectivity models were able to predict brain-age, a well-established surrogate biomarker. Here, we predicted age from the same input data as in the main analysis after first removing age from the input features set. In line with our expectations, functional connectivity increased predictive performance when combined with other modalities (e.g., in combination with non-brain, performance increased from 0.45 to 0.53; Fig. 4), and functional connectivity alone could predict age reasonably well (median R^2^ = 0.33, Figure S6), suggesting that its negligible contribution to decline prediction cannot be attributed to general methodological or data quality issues.

In the main analysis, the predictive target of cognitive decline was quantified as a continuous score. To compare our analysis to previous work that predicted classes of cognitive decline, we performed a further analysis that predicted extreme groups of cognitive decline (stable vs decline). Overall, extreme groups could be accurately predicted from the input data (most F1-scores [harmonic mean of the precision and recall] in the range of 0.8–0.9; Figure S7).

## 4. Discussion

In the present study, we found that combining baseline structural brain imaging data with nonbrain data improved the prediction of future cognitive decline. In contrast, functional connectivity features did not improve prediction. By predicting future cognitive decline as a continuous trajectory, rather than a diagnostic label, our study broadens the scope of applications to cognitive decline in healthy aging. It also allows for more nuanced predictions on an individual level. In the future, these continuous measures may facilitate dimensional approaches to pathology (Cuthbert, 2014).

The benefit of combining structural with nonbrain data found in the present study is well in line with previous work that predicted conversion from MCI to AD (Korolev et al., 2016), and classes of cognitive decline (Bhagwat et al., 2018). Nonbrain data alone predicted cognitive decline and the model was robustly improved by adding structural data (R2 increased from 0.36 to 0.42 for CDR and from 0.32 to 0.34 for MMSE). These findings are consistent with prior work (Korolev et al., 2016; Bhagwat et al. 2018). In general, the range of accuracies reported in our study is well in line with previous work predicting a related continuous target (time to symptom onset in AD) (Vogel et al. 2018), as well as with work predicting diagnostic labels (e.g., Eskildsen et al., 2015; Korolev et al., 2016; Gaser et al., 2013; Davatzikos et al., 2011). After having established that a combination of nonbrain and structural data gives predictions worthy of consideration, next, we assessed which features drove the predictions.

We found that clinical and neuropsychological assessments and subcortical structures drove the performance of our model. Measurements of memory, verbal fluency, executive function, and a wide set of cognitive and daily functions (MMSE, CDR, and FAQ) were the most informative nonbrain features for predicting cognitive decline. This matches well with Korolev et al., (2016) who found memory scores and clinical assessments (ADAS-Cog, FAQ) to be among the most informative nonbrain features. On the other hand, hippocampus and amygdala volume were the most informative structural features in our analysis, which is well in line with previous work predicting conversion from MCI to AD (Korolev et al., 2016; Eskildsen et al., 2015). In contrast, risk factors (such as age, APOE, or health risks) and markers that quantify general brain atrophy and regional cortical brain structure did not add markedly to model performance. It should be noted that features were assessed using permutation importance, which underestimates the importance of correlated features. Baseline MMSE and CDR-SOB scores are substantially correlated in the general population (Balsis et al., 2015) and are thus susceptible to this attenuation. We note, however, that both still figure among the top 15 features in Fig. 3. Alternative approaches, such as mean decrease impurity, might complement the permutation-based approach in future studies to improve the sensitivity (Engemann et al., 2020). Nevertheless, taken together, our results suggest that memory, everyday functioning, and subcortical features better predict future cognitive decline at the individual level than risk factors or global brain characteristics.

Functional connectivity, in contrast to brain structure, did not improve predictions when added to other modalities, nor did it predict cognitive decline on its own. While many previous studies predicted cognitive performance or decline based on structural imaging, studies using functional connectivity are rare and contain widely varying estimates of its predictive power (Dansereau et al., 2017; Hojjati et al., 2018; Vogel et al., 2018). Although functional connectivity in our study did not predict future cognitive decline, it did predict brain age. Assuming that functional connectivity is at least somewhat predictive of future cognitive decline, our analysis may suggest that the processing of functional connectivity data was not a good fit for the cognitive targets. Furthermore, data with better spatial and temporal resolution might be able to better capture decline. This calls for future studies that benchmark different processing options as these can severely impact predictive accuracy (Dubois et al., 2018).

In the following, we will sketch possible future developments along 4 themes: implications of and possible improvements to the continuous *targets of cognitive decline, multimodal input data, predictive models*, and the importance of *generalization* to new datasets.

Quantifying cognitive decline continuously rather than discretely enables a more fine-grained and robust prediction, but also requires methodological choices. By predicting a diagnostic label, previous studies were often restricted to MCI patients and aimed to distinguish stable from converting patients. Considering decline as a continuum better characterizes the underlying change in abilities and allows for capturing changes that occur in healthy aging. Overcoming the scarcity of diagnosed conditions, widens the scope of applications and has methodological advantages: the resulting increased sample size yields more robust models, which is critical to avoiding optimistic bias in estimating prediction accuracy (Woo et al., 2017; Varoquaux, 2018). Furthermore, our approach also does not require assigning a diagnostic label, which entails subjective clinical judgment and arbitrary cut-off values. Considering cognitive decline as a continuous target does, however, require a model to aggregate multiple longitudinal measurements. Here, we used participant-specific linear slopes estimated through longitudinal data from clinical assessments. Since cognitive decline also shows nonlinear trajectories (Wilkosz et al., 2010), one could argue that accounting for nonlinearity is called for when extracting the predictive targets. However, robustly estimating nonlinearity requires more longitudinal measurements per participant and more complex models. In contrast, linear trajectories can robustly be estimated with 3 measurements, hence, they provide a useful approximation of cognitive decline. Notably, the baseline values of the clinical assessments used to define the slopes have a special role: they are input features and the slopes are defined relative to them. This might result in a bias due to regression to the mean (Barnett, van der Pols, and Dobson, 2005), where unusually extreme baseline values (due to noise) might result in unusually extreme slopes (returning to the mean). This issue is relevant as well when defining diagnostic labels where it might result in patients switching between labels due to noise. Future studies should consider more complex models that can better account for these effects. Taken together, quantifying cognitive decline continuously allows for a more nuanced representation of decline and widens the scope of applications. However, while refining the definition of cognitive decline is warranted, it requires more complex analytical approaches and appropriate data.

In this study, we quantified cognitive decline using 2 clinical assessments (CDR and MMSE), which measure a heterogeneous set of cognitive and everyday life functions. While these clinical assessments have the advantage of being used in practice, they lack the specificity to target single cognitive constructs. Measuring cognitive constructs more homogeneously might potentially improve accuracy, especially if those constructs are strongly linked to specific brain regions or networks. This could be achieved by additionally employing neuropsychological assessments. The multitarget approach outlined in this study is well-suited to including these additional targets.

Beside additional targets, future studies should also consider additional multimodal input data to characterize the brain in greater detail. The present study used data derived from structural and functional MRI (T1w and resting-state fMRI). These might be complemented by information from diffusion-weighted imaging, arterial spin labeling, or positron emission tomography (Rahim et al., 2016). Additionally, the presently used modalities could also be refined and alternative representations could be considered. For instance, different methods for quantifying brain structure (Pipitone et al., 2014) or brain function (Rahim, Thirion, and Varoquaux, 2019), and adding data on structural asymmetry (Wachinger et al., 2016) or dynamic functional connectivity (Filippi et al., 2019) could provide improved predictive performance. Furthermore, the influence of MR data quality on accuracy should be assessed in future studies. While our past work showed that brain-age prediction from multimodal neuroimaging is robust against in-scanner head motion (Liem et al., 2017), the present study has not assessed the influence of MR data quality on predictive accuracy. Addressing this issue would yield recommendations regarding the required data quality to predict cognitive decline.

The predictive approach could also be expanded to better accommodate high-dimensional data and the messiness of real-world data acquisition. The present study concatenated low-dimensional features across modalities and fed them into 1 random forest model. Including all features in 1 model allowed us to consider feature-level interactions across modalities. Alternatively, *prediction stacking* could be used to facilitate the integration of multimodal data (Liem et al., 2017; Rahim et al., 2016; Engemann et al., 2020). While the stacking approach accounts for modality-level interactions it does not consider feature-level interactions across modalities. However, it scales well to high-dimensional data and allows for block-wise missing data, for instance, a missing modality. The present work only included participants if data from all modalities (nonbrain, structural, functional) were available. In clinical practice, this is often not feasible. As we demonstrated previously, stacking can be used to include participants with missing modalities, which increases the sample size and the scope of application (Engemann et al., 2020).

In practice, the benefit of adding multimodal neuroimaging data to a set of clinical assessments needs to be considered against the additional costs. Its clinical utility also depends on the actionable insight that can be drawn from an earlier prognosis. Of course, this concern is not specific to this study; it applies broadly to almost every effort to incrementally predict clinically meaningful outcomes from brain-based measures. At the moment, no causal treatment for cognitive decline is available. However, an early prognosis might aid intervention studies and be even more helpful once effective treatments are available. Hence, future studies should further exploit the information yielded by the model to focus on participant-specific predictions. In general, predictive models don’t perform equally well in all circumstances. For some participants or sub-groups, a more confident prediction is possible. Recent work demonstrated a higher prediction accuracy in participants with certain characteristics, for example, older, female, etc. (Korolev et al., 2016). This enables increased accuracy by focusing on high-confidence predictions (Tam et al., 2019) and might even suggest a participant-tailored clinical workflow depending on the prediction confidence (Bhagwat et al., 2018). While the present study has not yet investigated these effects, it is well set up to determine optimal conditions for model performance. A large number of cross-validation splits yields a distribution of predictive performance, not only a point estimate. This will also allow us to assess whether the predictions across sub-groups are driven by the same features.

For a predictive model to be useful in real-world applications, it needs to generalize well to datasets from different sites (Scheinost et al., 2019). While characteristics of our study facilitate generalization, a future study is required to empirically establish the generalization of our models to independent datasets. First, we have aimed to provide full transparency throughout this study to improve reproducibility and generalizability. We used data from a large, publicly available dataset, preprocessed them with well-established open-source tools, and inputted them into well-established models. The analysis code is publicly shared and after further developing this approach, trained models will also be shared. Importantly, the analysis was preregistered to avoid overfitting due to analytical flexibility (Carp, 2012; Hosseini et al., 2020). Second, the OASIS-3 project is set up heterogeneously regarding the number of sessions, the intervals between sessions, and the participants’ duration in the study. This heterogeneity is expected to provide less opportunity for an algorithm to overfit to dataset-specific idiosyncrasies, resulting in more generalizable models that also perform well in other settings.

While a heterogeneous dataset and open/reproducible approaches certainly improve generalizability, we trained and tested models using only one dataset. Thus, the cross-validated performance in our study provides a biased estimate of the generalizability to independent datasets. This bias might even be modality-specific, in that non-brain features might generalize better than brain imaging features (Bhagwat et al., 2018). Training predictive models on data from multiple sites has been shown to improve generalization (Abraham et al., 2017; Orban et al., 2018; Liem et al., 2017). Hence, future studies should use models trained and tested on data from multiple sites, which requires further suitable longitudinal and publicly available datasets (Varoquaux, 2018). This also provides an opportunity to take preregistration even further. After conducting experiments in an initial dataset, a trained model could be preregistered and applied to an independent dataset that hasn’t yet been analyzed.

Future research could investigate the impact of different preprocessing strategies on predictive performance. This encompasses everything from individual settings to the software used itself. For example, on the volumetry of subcortical structures, substantial differences are noted between different software (Mulder et al., 2014; Bartel et al., 2017).

## 5. Conclusions

In summary, we have shown that adding structural brain imaging data to nonbrain data (such as memory scores or everyday functioning) improves the prediction of future cognitive decline in healthy and pathological aging. Conversely, adding functional connectivity data, as used in the present approach, did not aid the prediction. Importantly, our work has potential for clinical utility by predicting *future* cognitive decline, rather than a *current* diagnosis. Future studies should include additional brain imaging modalities and independent datasets and should determine the potential of functional connectivity using alternative methodological approaches. Quantifying future decline continuously allows for more nuanced predictions on an individual level. In the future, these continuous measures may facilitate dimensional approaches to pathology (Cuthbert, 2014)

Increased personal and societal costs due to healthy and pathological age-related cognitive decline are one of the most pressing challenges in an aging society. An early and individually fine-grained prognosis of age-related cognitive decline allows for earlier and individually targeted behavioral, cognitive, or pharmacological interventions. Intervening early increases the chances to attenuate or prevent cognitive decline, which will alleviate both personal and societal costs. Importantly, our work targets applications to healthy aging, widening the scope beyond the pathological to the entire aging population.

## Supplementary Material

1

## Figures and Tables

**Table 1:** Comparison of a nonexhaustive selection of studies that perform prediction of future cognitive decline in the context of AD. Compared with the literature, which is often concerned with predicting discrete class assignment, our approach predicts rates of cognitive decline based on a wide selection of input features.

	Targets	Inputs	Analysis method

(Eskildsen et al., 2015)	MCI to AD conversion	Regional cortical thickness, nonlocal hippocampal morphological grading scores, clinical scores (MMSE, RAVLT), age	Linear discriminant analysis with multivariate feature selection
(Korolev et al., 2016)		Clinical scores (risk factors, clinical assessments, medication status), regional GM morphometry, (cortical and subcortical volumes, mean cortical thickness, standard deviation of cortical thickness, surface area, curvature), plasma proteomics biomarkers	Probabilistic multiple kernel learning (pMKL) with multivariate feature selection
(Gaser et al., 2013)		Estimated “brain age” score, baseline clinical scores, age, hippocampal volume	Cox regression, ROC analysis
Davatzikos et al., 2011)		Automated marker of atrophy (SPARE-AD), CSF biomarkers	SVM
(Bhagwat et al., 2018)	Membership to clusters of MMSE and ADAS-13 trajectories	Regional cortical thickness, APOE4 status, age and baseline clinical scores	Longitudinal siamese network
Current study	Rate of MMSE and CDR-SOB change	Mean cortical thickness, GM, WM and CSF total volumes, regional subcortical volumes, functional connectivity, age, APOE status, baseline clinical scores, demographic information, health status, neuropsychological assessment	Multitarget Random Forest

**Table 2:** Sample characteristics. N = 662 (302 male). See Table 3 for a list of abbreviations

	M	SD	min	max	N missing

Demographics					
Age _baseline_	71	8.2	46	96	
Sex	302 M;360 F				
Years of education _baseline_	15	2,7	7	29	11
Clinical scores					
MMSEb _baseline_	28	2.2	16	30	
CDR-SOB _baseline_	0.62	1.37	0.00	8.00	
FAQ _baseline_	1.35	3.38	0.00	23.0	14
NPI-Q					
Presence_baseline_	0.95	1.64	0.00	10.0	15
Severity_baseline_	1.4	2.8	0.0	18	15
GDS_baseline_	1.5	2.0	0.0	12	17
Genotyping (APOE alleles)					
E2 count	0: 565; 1: 91; 2: 5				1
E3 count	0: 61; 1: 272; 2: 328				1
E4 count	0: 403; 1: 223; 2: 35				1
Diagnostic and sessions					
Clinical Diagnosis_baseline_	509 HC; 12 MCI; 111 DE				30
N clinical sessions	5.8	2.4	3	15	
Years between clinical	1.2	0.6	0.003	5.5	
sessions					
Years in study	5.8	2.5	1.6	10.9	
Outcomes					
MMSE slope (1/y)	−0.31	0.93	−7.5	2.9	
CDR-SOB slope (1/y)	0.25	0.61	−1.3	4,4	

**Table 3:** List of abbreviations of clinical tests.

CDR-SOB	Clinical Dementia Rating - Sum of Boxes

FAQ	Functional Activities Questionnaire
GDS	Geriatric Depression Scale
MMSE	Mini-Mental State Examination
NPI-Q	Neuropsychiatric Inventory Questionnaire
TMT	Trail Making Test
WAIS-R	Wechsler Adult Intelligence Scale-Revised
WMS-R	Wechsler Memory Scale-Revised
BNT	Boston Naming Test

## Footnotes

1 The selected participants and session can be found here: https://github.com/fliem/cpr/tree/0.1.2/info.

2 https://github.com/NrgXnat/oasis-scripts.

3 https://central.xnat.org/.

4 http://github.com/fliem/cpr.

5 https://hub.docker.com/r/fliem/cpr.
