# Supplementary material for: Predicting future cognitive decline from non-brain and multimodal brain imaging data in healthy and pathological aging
Source: Neurobiol Aging. Author manuscript; Available in PMC 2023 Jan 20. (PMC9853405; doi:10.1016/j.neurobiolaging.2022.06.008)
Supplement: 1 [file NIHMS1861086-supplement-1.pdf]

Predicting future cognitive decline from non-brain and multimodal brain imaging data in healthy and pathological aging

6 Appendix

6.1 Supplementary Methods

6.1.1 Details on MRI acquisition

Acquisition Scanner	Voxel-size (mm x mm x mm)	TR (ms)	TE (ms)	Sessions/ subjects
T1wanatomical				
TrioTim	1x1x1	2400	31	60/30
	1x1.02x1.02	2400	31	1/1
	1x1x1	2400	32	1010/590
Biograph mMR	1x1x1	2400	21	2/2
	1x1.05x1.05	2300	30	44/42
Missing	1x1x1	missing	missing	2/1
T2wanatomical				
TrioTim	1x1x1	3200	454	2/2
	1x1x1	3200	455	616/616
RS-fMRI				
TrioTim	4x4x4	2500	27	11/7
	4x4x4	2200	30	8/8
	3.3x3.3x3.3	2200	27	1228/625
Biograph mMR	4x4x4	2200	27	80/40
Missing	missing	missing	missing	4/1

Table S1 Number of scans by parameter combination. TR for fMRI signifies the time-resolution, while for T1w and T2w it is the time between excitation pulses. Only fMRI scans lasting more than 2 minutes were kept. All 662 subjects had at least one fMRI scan lasting at least 6 minutes.

6.1.1 Details on MRI preprocessing[1]

Results included in this manuscript come from data preprocessed using *fMRIPrep* 1.4.1 (RRID:SCR_016216; Esteban, Blair, et al. 2018; Esteban, Markiewicz, et al. 2018), which is based on *Nipype* 1.2.0 (RRID:SCR_002502; K. Gorgolewski et al. 2011; K. J. Gorgolewski et al. 2018).

6.1.1.1 Anatomical data preprocessing

T1-weighted (T1w) images were corrected for intensity non-uniformity (INU) with `N4BiasFieldCorrection` (Tustison et al. 2010), distributed with *ANTs* 2.2.0 (RRID:SCR_004757; Tustison et al. 2010; Avants et al. 2008). The T1w-reference was then skull-stripped with a *Nipype* implementation of the `antsBrainExtraction.sh` workflow (from *ANTs*), using OASIS30ANTs as target template. Brain tissue segmentation of cerebrospinal fluid (CSF), white-matter (WM) and gray-matter (GM) was performed on the brain-extracted T1w using `fast` (*FSL* 5.0.9) (RRID:SCR_002823; Tustison et al. 2010; Avants et al. 2008; Zhang, Brady, and Smith 2001). A T1w-reference map was computed after registration of the T1w images (after INU-correction) using `mri_robust_template` (*FreeSurfer* 6.0.1) (Reuter, Rosas, and Fischl 2010). Brain surfaces were reconstructed using `recon-all` (*FreeSurfer* 6.0.1) (RRID:SCR_001847; Reuter, Rosas, and Fischl 2010; Dale, Fischl, and Sereno 1999), and the brain mask estimated previously was refined with a custom variation of the method to reconcile *ANTs*-derived and *FreeSurfer*-derived segmentations of the cortical gray-matter of Mindboggle (RRID:SCR_002438; Klein et al. 2017). Volume-based spatial normalization to one standard space (MNI152NLin2009cAsym) was performed through nonlinear registration with `antsRegistration` (*ANTs* 2.2.0), using brain-extracted versions of both T1w reference and the T1w template. The following template was selected for spatial normalization: *ICBM 152 Nonlinear Asymmetrical template version 2009c* (Fonov et al. 2009) (RRID:SCR_008796, TemplateFlow ID: MNI152NLin2009cAsym). Where available, T2w-images were included for surface reconstruction.

6.1.1.2 Functional data preprocessing

For each BOLD run, the following preprocessing was performed. First, a reference volume and its skull-stripped version were generated using a custom methodology of *fMRIPrep*. The BOLD reference was then co-registered to the T1w reference using `bbregister` (*FreeSurfer*) which implements boundary-based registration (Greve and Fischl 2009). Co-registration was configured with nine degrees of freedom to account for distortions remaining in the BOLD reference. Head-motion parameters with respect to the BOLD reference (transformation matrices, and six corresponding rotation and translation parameters) are estimated before any spatiotemporal filtering using `mcflirt` (*FSL* 5.0.9) (Jenkinson et al. 2002). The BOLD time-series (including slice-timing correction) were resampled onto their original, native space by applying a single, composite transform to correct for head-motion and susceptibility distortions. These resampled BOLD time-series will be referred to as *preprocessed BOLD in original space*, or just *preprocessed BOLD*. The BOLD time-series were resampled into standard space, generating a *preprocessed BOLD*

[1] The description in this section was automatically created by *fMRIPrep* and adapted where needed.

run in MNI152NLin2009cAsym space. Several confounding time-series were calculated based on the *preprocessed BOLD*: framewise displacement (FD), DVARS and three region-wise global signals. FD and DVARS are calculated for each functional run, both using their implementations in *Nipype* (following the definitions by (Power et al. 2014). The three global signals are extracted within the CSF, the WM, and the whole-brain masks.

All resamplings can be performed with *a single interpolation step* by composing all the pertinent transformations (i.e. head-motion transform matrices, susceptibility distortion correction when available, and co-registrations to anatomical and output spaces). Gridded (volumetric) resamplings were performed using `antsApplyTransforms` (*ANTs*), configured with Lanczos interpolation to minimize the smoothing effects of other kernels (Lanczos 1964). Many internal operations of *fMRIPrep* use *Nilearn* 0.5.2 (RRID:SCR_001362; Abraham et al. 2014), mostly within the functional processing workflow. For more details of the pipeline, see the section corresponding to workflows in *fMRIPrep's* documentation[2].

6.1.2 Deviation from preregistration

This analysis has been preregistered (Liem et al. 2019). While we have largely followed the plan, the analysis deviates in several minor points:

- Subjects were only considered for the study if they had at least three clinical sessions (not two as preregistered). Extracting the slopes of cognitive decline from two sessions resulted in very noisy slopes. As a result, the sample size is not 849 as preregistered, but 662. As our learning curve experiments demonstrate, the resulting sample size is sufficient in the present context.
- After further discussion, we added two features that have not been preregistered to the non-brain feature set: i) the number of sessions prior to the baseline session (to account for retest effects), and ii) the cognitive diagnosis at baseline (healthy, mild cognitive impairment, dementia). Both features did not show high importance in the permutation importance analysis.
- The preregistered structural features set included 331 features from global, subcortical, and cortical (volume and thickness) markers. After finding that the reduced structGS feature set (35 global and subcortical markers) performs equally well, we conducted the analyses with the more parsimonious structGS modality.
- After further discussion, the preregistered dimensionality reduction approach for the functional connectivity data seemed suboptimal. It averages positive and negative values, which might result in the cancellation of positive and negative connectivity within a network. The updated approach downsampled the connectivity matrix using a PCA.
- In the preregistration, two hyperparameters were planned to be used for tree pruning (max_depth = [5, 10, 20, 40, 50, None], where None leads to fully grown trees and min_samples_leaf = [1, 4, 10]). After further discussion, we decided to remove this double parametrization and only tune max_depth. Additionally, a currently ongoing independent project suggested that lower values of max_depth might be worth investigating in more detail. Hence, we added, max_depth = [3, 7, 15] to the hyperparameter tuning. Since the hyperparameters did not have a large influence on

[2] https://fmriprep.readthedocs.io/en/latest/workflows.html

the result and those added values did not render the best performance we deem this change inconsequential.

- The results of the main analysis suggested further validation analyses (brain-age, extreme group classification). Those were not preregistered. However, they are very similar to the main analysis (only the models were adapted to the question: ridge regression for the brain-age analysis; random forest classifier for the extreme group classification with the criterion to measure the quality of an RF-split tuned with ["gini", "entropy"]).

6.1.3 List of features

Modality	# features modality	Name	Test/Variables	Information	# features
**non-brain**	66	demographic information	Age, sex, education		3
		clinical assessments	MMSE	Sum score	1
			CDR	6 items, global and SOB scores	8
			FAQ	10 items and sum score	11
			NPI-Q	Symptom presence and severity sum scores	2
			GDS	Sum score	1
		neuropsychology	WMS-R	Logical memory (LOGIMEM, MEMUNITS, MEMTIME) Digit span (DIGIF, DIGIFLEN, DIGIB, DIGIBLEN)	7
			Word fluency	ANIMALS, VEG	2
			TMT	Part A (TRAILA, TRAILARR, TRAILALI), Part B (TRAILB, TRAILBRR, TRAILBLI), TRAILBnorm = TRAILB/TRAILA	7
			WAIS-R	Digit Symbol	1
			BNT	BOSTON	1
		APOE	ε2, ε3, and ε4 allele count		3
		cognitive diagnosis		healthy control, MCI, or dementia	1
		health information	cardio/cerebro-vascular health, diabetes, hypercholesterolemia, smoking, and family history of dementia		17
		N sessions before baseline			1
**structGS**	35	subcortical volume	accumbens, amygdala, caudate, hippocampus, pallidum, putamen, thalamus	Volume of left and right	14
		global measurements	L + R mean cortical thickness; L + R lateral, 3rd, 4th ventricles; L + R total cortical volume; L + R cerebral white matter volume; L + R cerebellar white matter and cortical volume; total subcortical gray matter volume, total gray matter volume, corpus callosum volume (5 parcels)		21
**func**	100	functional connectivity	300 cortical, cerebellar, and subcortical ROIs	reduced to 100 PCA components	100

Table S2. Input features

6.2 Supplementary Results

6.2.1 Predictive targets

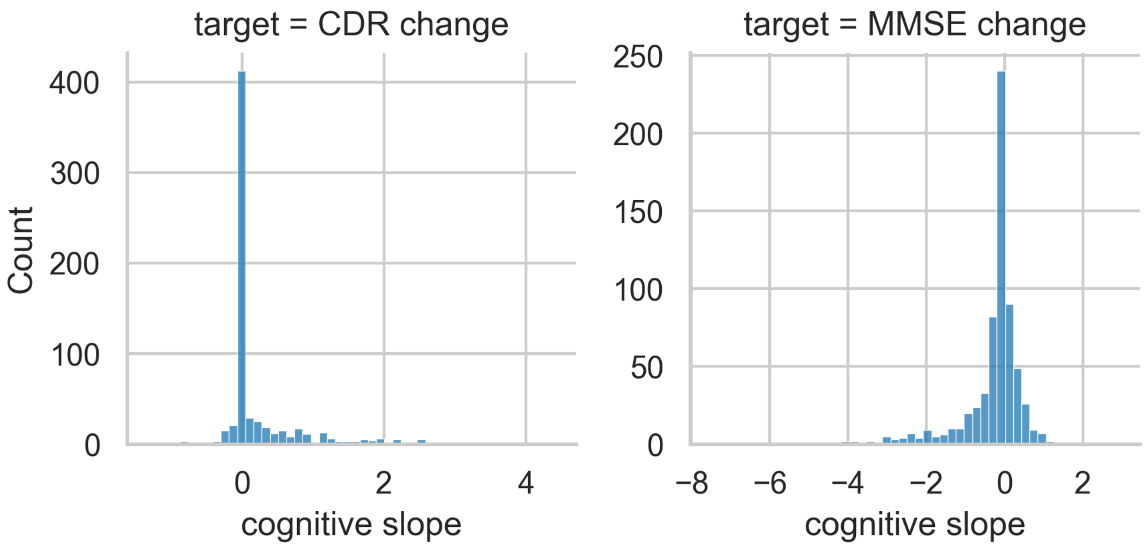

Figure S1. Distribution of predictive targets (cognitive slopes of CDR and MMSE).

6.2.2 Predictive performance (full results)

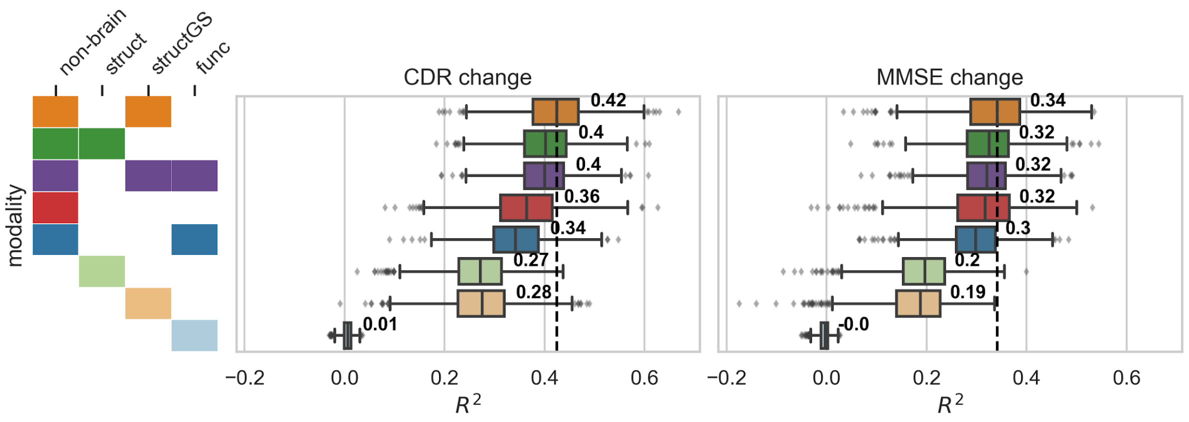

Figure S2. Adding structural data to non-brain data improves prediction of cognitive decline. Test performance (R^2, coefficient of determination) across splits. Targets: cognitive decline measured via CDR (Clinical Dementia Rating, top) and MMSE (Mini-Mental State Examination, bottom). Input modalities: non-brain, structGS (global and subcortical structural volumes), struct (structGS + cortical volume and thickness), func (functional connectivity). Left panel represents combinations of input modalities (e.g., first line is non-brain + structGS). The number represents the median, the dashed vertical line marks the median of the best-performing combination of modalities (within a target). This figure is an extension of Figure 2 and also includes brain modalities on their own.

modality / target	outperforming non-brain (% of splits) CDR change	MMSE change	mean difference CDR change	MMSE change	SD difference CDR change	MMSE change
structGS	19	5	-0.09	-0.13	0.1	0.09
struct	17	7	-0.09	-0.12	0.1	0.09
func	0	0	-0.36	-0.31	0.08	0.08
non-brain + structGS	92	78	0.06	0.02	0.04	0.04
non-brain + struct	75	59	0.04	0.01	0.06	0.05
non-brain + func	25	31	-0.02	-0.01	0.03	0.03
non-brain + structGS + func	78	57	0.04	0.01	0.05	0.04

Table S3. Comparison of test performance vs non-brain predictions (% of splits for which the test prediction R^2 is outperforming the non-brain prediction; N_{splits} = 1000). Mean and SD difference show the mean and standard deviation of the modality's performance vs. the non-brain prediction. Best performing model (gray): the CDR prediction from *non-brain + structGS* is outperforming the non-brain prediction in 91% of splits.

modality / target	outperforming null-models (% of splits) CDR change	MMSE change	median difference CDR change	MMSE change	SD difference CDR change	MMSE change
non-brain	100	100	0.42	0.36	0.09	0.09
structGS	100	99	0.31	0.21	0.08	0.07
struct	100	100	0.34	0.24	0.08	0.07
func	91	73	0.01	0.01	0.01	0.01
non-brain + structGS	100	100	0.48	0.39	0.08	0.09
non-brain + struct	100	100	0.47	0.38	0.09	0.09
non-brain + func	100	100	0.42	0.35	0.08	0.07
non-brain + structGS + func	100	100	0.47	0.37	0.08	0.08

Table S4. Comparison of test performance vs null-models (% of splits for which the test prediction R^2 is outperforming the null-model prediction). Best performing model (gray): the CDR prediction from *non-brain + structGS* is outperforming the non-brain prediction in 100% of splits.

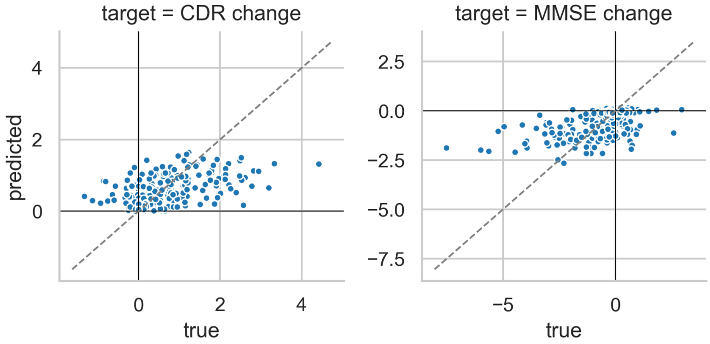

Figure S3. Scatter plot of true vs predicted trajectories of cognitive change for the *non-brain + structGS* model. Mean prediction across splits. CDR: positive values = cognitive decline, MMSE: negative values = cognitive decline.

6.2.3 Hyperparameter tuning of the random forest regression model

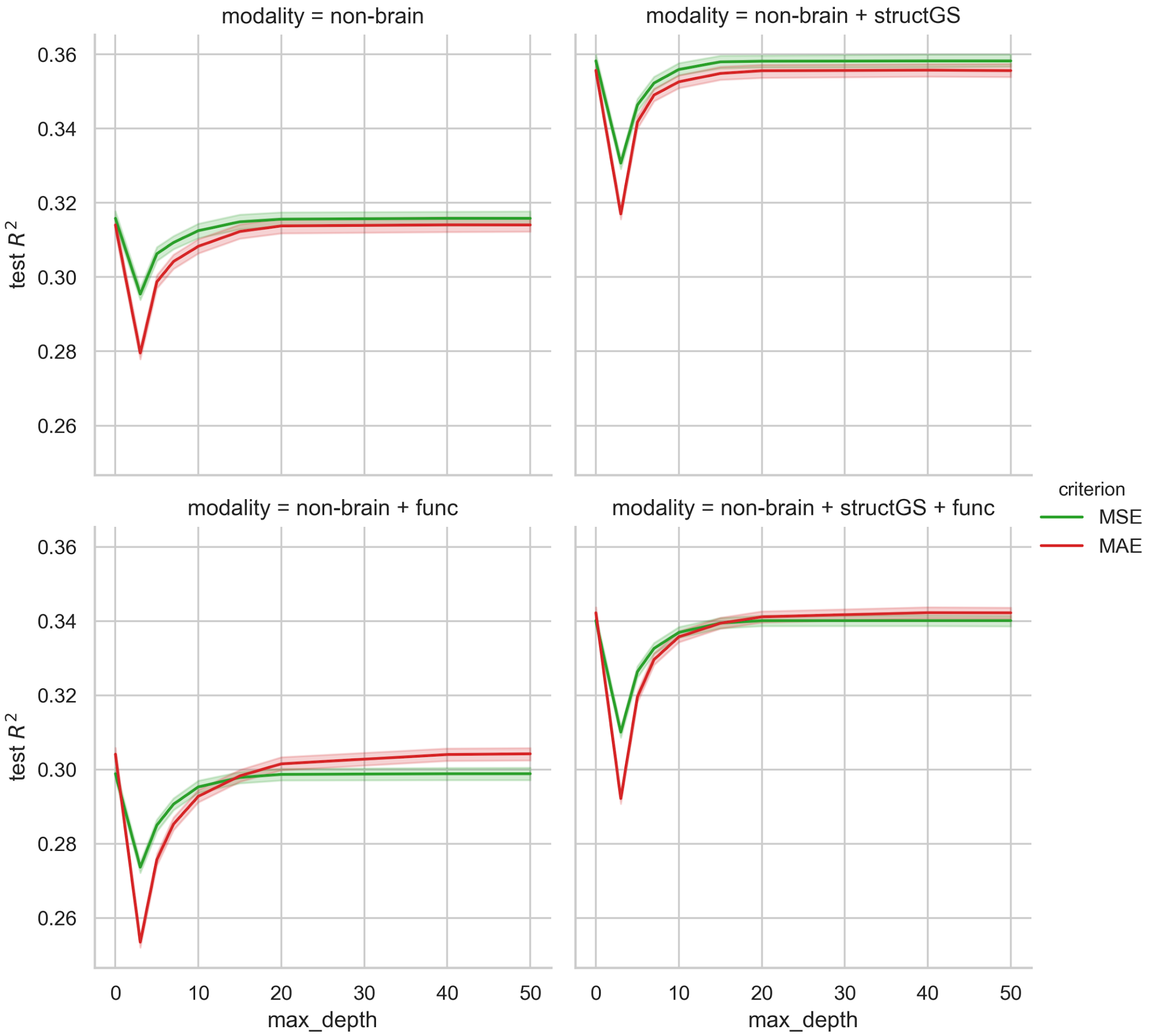

Figure S4. Tuning curves of the random forest regression hyperparameters max tree depth (0 represents fully grown trees) and criterion (MSE: mean squared error, MAE: mean absolute error). Note that the y-axis is trimmed and the results vary in a rather narrow range.

6.2.4 Learning curves demonstrate sufficient sample size

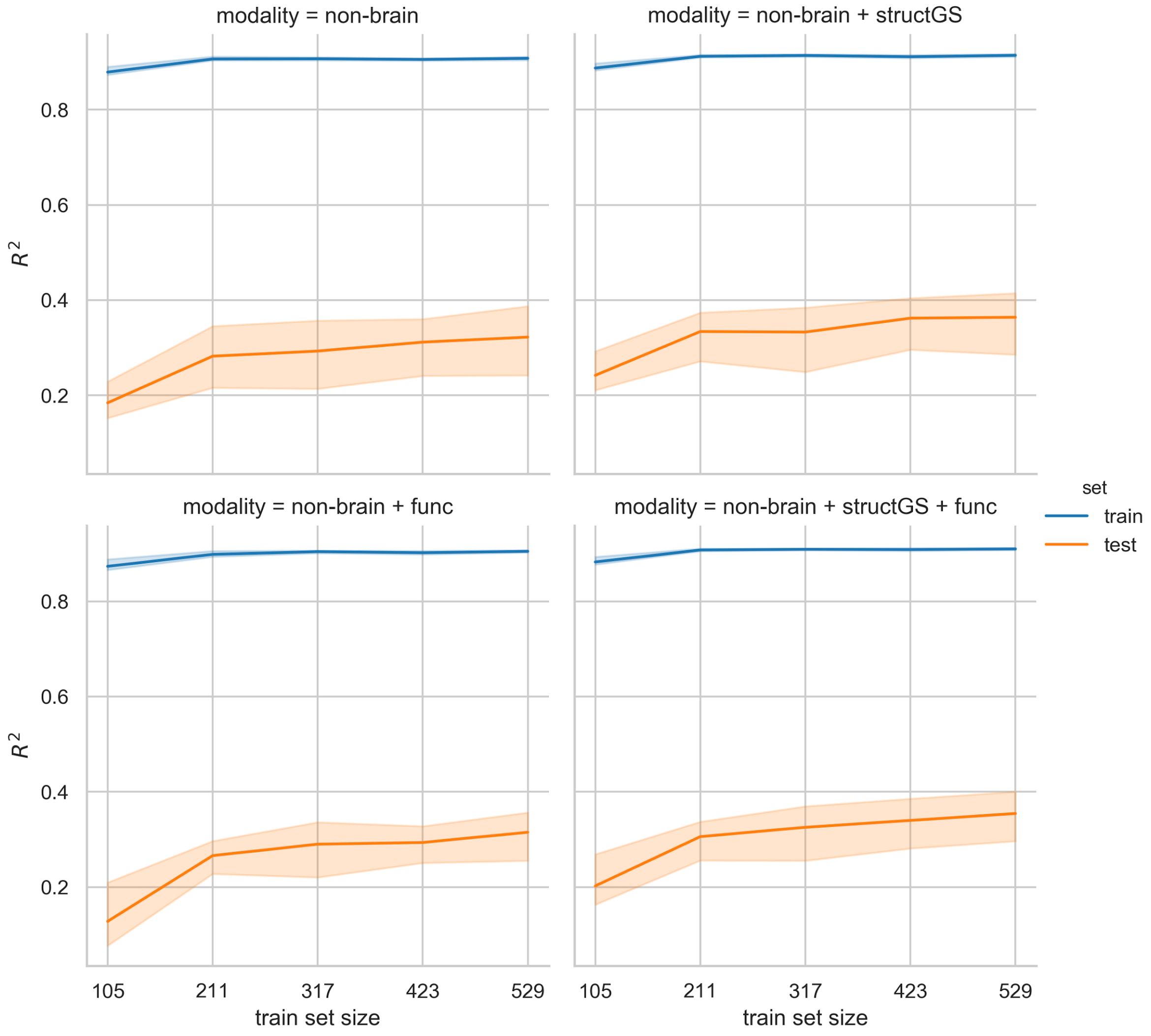

Figure S5. Learning curve plotting the models' performance across an increasing training sample size.

6.2.5 Age prediction

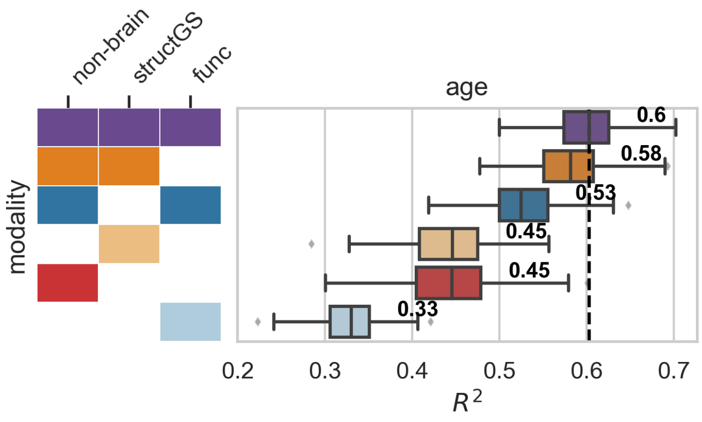

Figure S6. Predictive performance in brain-age prediction. Input modalities: non-brain, structGS (global and subcortical structural volumes), func (functional connectivity). The number represents the median, the dashed vertical line marks the median of the best-performing combination of modalities. This figure is an extension of Figure 4 and also includes brain modalities on their own.

6.2.6 Extreme group prediction

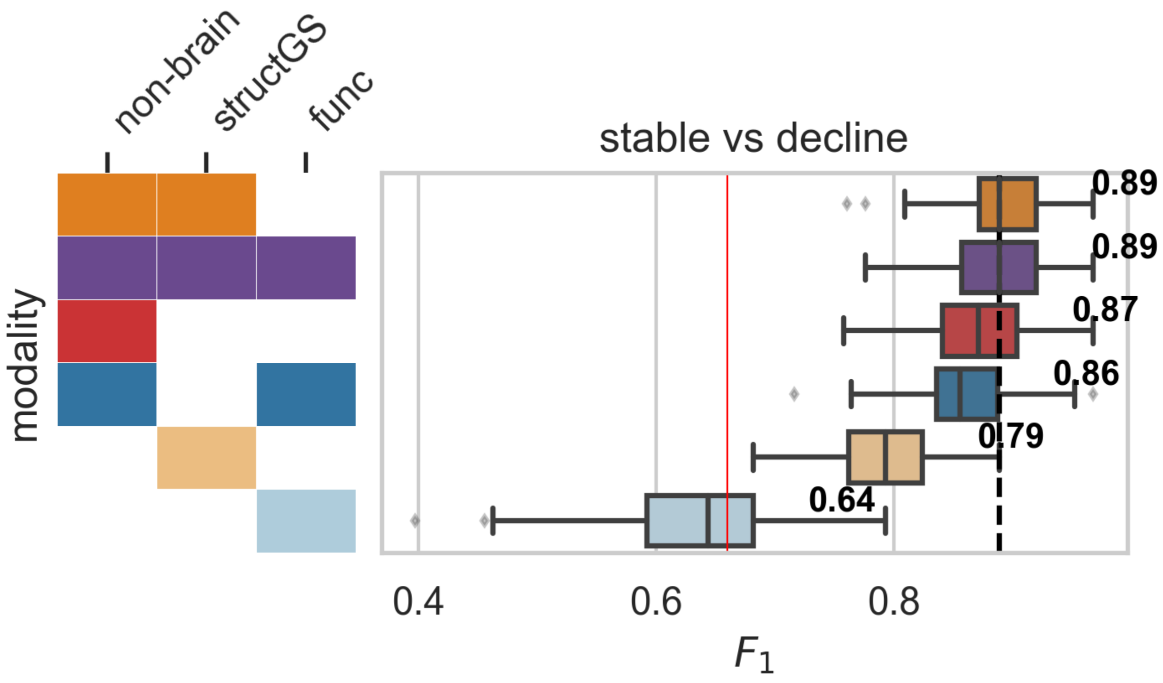

Figure S7. Predictive performance in predicting extreme groups of cognitive decline (stable vs decline; F1-score: harmonic mean of the precision and recall). Input modalities: non-brain, structGS (global and subcortical structural volumes), func (functional connectivity). The number represents the median, the dashed vertical line marks the median of the best-performing combination of modalities, the red solid line represents the non-informative baseline score. For this scenario, the baseline score was calculated to be 0.66. See supplementary text for a derivation.

A baseline F1-score for binary classification can be readily derived. Starting from the definition of the F1-score, with precision P and recall R

$F_1 = 2 \cdot P \cdot R / (P + R)$

If we randomly assign the positive class based on a coin-flipping procedure with probability q, and our sample has positive incidence p and n total samples, we can show that (see Grüne 2020).

$TP = npq,\ TN = n(1 - q)(1 - p),\ FP = n(1 - p)q\ ,\ FN = np(1 - q)$

$P = TP/(TP + FP) = npq/(npq + n(1 - p)q) = p$

$R = FP/(TP + FN) = npq/(npq + np(1 - q)) = q$

Therefore, for this baseline score,

$F_1 = 2 \cdot p \cdot q / (p + q)$

Under balanced classes, as in our example, $p = 0.5$

$F_1 = q / (0.5 + q)$

Under this non-informative regime, F1 is maximized when $q = 1$, i.e., all samples are assigned to the positive class, resulting in

$F_1 = 1 / (0.5 + 1) = 1/1.5 = 0.66$

7 References

Abraham, Alexandre, Fabian Pedregosa, Michael Eickenberg, Philippe Gervais, Andreas Mueller, Jean Kossaifi, Alexandre Gramfort, Bertrand Thirion, and Gael Varoquaux. 2014. “Machine Learning for Neuroimaging with Scikit-Learn.” *Frontiers in Neuroinformatics* 8. https://doi.org/10.3389/fninf.2014.00014.

Avants, B. B., C. L. Epstein, M. Grossman, and J. C. Gee. 2008. “Symmetric Diffeomorphic Image Registration with Cross-Correlation: Evaluating Automated Labeling of Elderly and Neurodegenerative Brain.” *Medical Image Analysis* 12 (1): 26–41.

Dale, Anders M., Bruce Fischl, and Martin I. Sereno. 1999. “Cortical Surface-Based Analysis: I. Segmentation and Surface Reconstruction.” *NeuroImage* 9 (2): 179–94.

Esteban, Oscar, Ross Blair, Christopher J. Markiewicz, Shoshana L. Berleant, Craig Moodie, Feilong Ma, Ayse Ilkay Isik, et al. 2018. “fMRIPrep.” *Software: Practice & Experience*. https://doi.org/10.5281/zenodo.852659.

Esteban, Oscar, Christopher Markiewicz, Ross W. Blair, Craig Moodie, Ayse Ilkay Isik, Asier Erramuzpe Aliaga, James Kent, et al. 2018. “fMRIPrep: A Robust Preprocessing Pipeline for Functional MRI.” *Nature Methods*. https://doi.org/10.1038/s41592-018-0235-4.

Fonov, V. S., A. C. Evans, R. C. McKinstry, C. R. Almli, and D. L. Collins. 2009. “Unbiased Nonlinear Average Age-Appropriate Brain Templates from Birth to Adulthood.” *NeuroImage* 47, Supplement 1: S102.

Gorgolewski, K., C. D. Burns, C. Madison, D. Clark, Y. O. Halchenko, M. L. Waskom, and S. Ghosh. 2011. “Nipype: A Flexible, Lightweight and Extensible Neuroimaging Data Processing Framework in Python.” *Frontiers in Neuroinformatics* 5: 13.

Gorgolewski, Krzysztof J., Oscar Esteban, Christopher J. Markiewicz, Erik Ziegler, David Gage Ellis, Michael Philipp Notter, Dorota Jarecka, et al. 2018. “Nipype.” *Software: Practice & Experience*. https://doi.org/10.5281/zenodo.596855.

Greve, Douglas N., and Bruce Fischl. 2009. “Accurate and Robust Brain Image Alignment Using Boundary-Based Registration.” *NeuroImage* 48 (1): 63–72.

Grüne, Ansgar. 2020. “What Is a Good F1 Score? —.” Inside GetYourGuide. October 13, 2020. https://inside.getyourguide.com/blog/2020/9/30/what-makes-a-good-f1-score.
Jenkinson, Mark, Peter Bannister, Michael Brady, and Stephen Smith. 2002. “Improved Optimization for the Robust and Accurate Linear Registration and Motion Correction of Brain Images.” *NeuroImage* 17 (2): 825–41.
Klein, Arno, Satrajit S. Ghosh, Forrest S. Bao, Joachim Giard, Yrjö Häme, Eliezer Stavsky, Noah Lee, et al. 2017. “Mindboggling Morphometry of Human Brains.” *PLoS Computational Biology* 13 (2): e1005350.
Lanczos, C. 1964. “Evaluation of Noisy Data.” *Journal of the Society for Industrial and Applied Mathematics Series B Numerical Analysis* 1 (1): 76–85.
Liem, Franziskus, Pierre Bellec, Cameron Craddock, KamalakerDadi, Jessica S. Damoiseaux, Daniel S. Margulies, Christopher J. Steele, Gaël Varoquaux, and Tal Yarkoni. 2019. “Predicting Future Cognitive Change from Multiple Data Sources (pilot Study).” OSF. osf.io/gynbj.
Power, Jonathan D., Anish Mitra, Timothy O. Laumann, Abraham Z. Snyder, Bradley L. Schlaggar, and Steven E. Petersen. 2014. “Methods to Detect, Characterize, and Remove Motion Artifact in Resting State fMRI.” *NeuroImage* 84 (Supplement C): 320–41.
Reuter, Martin, Herminia Diana Rosas, and Bruce Fischl. 2010. “Highly Accurate Inverse Consistent Registration: A Robust Approach.” *NeuroImage* 53 (4): 1181–96.
Tustison, N. J., B. B. Avants, P. A. Cook, Y. Zheng, A. Egan, P. A. Yushkevich, and J. C. Gee. 2010. “N4ITK: Improved N3 Bias Correction.” *IEEE Transactions on Medical Imaging* 29 (6): 1310–20.
Zhang, Y., M. Brady, and S. Smith. 2001. “Segmentation of Brain MR Images through a Hidden Markov Random Field Model and the Expectation-Maximization Algorithm.” *IEEE Transactions on Medical Imaging* 20 (1): 45–57.
